# The endosymbiont *Wolbachia* rebounds following antibiotic treatment

**DOI:** 10.1371/journal.ppat.1008623

**Published:** 2020-07-08

**Authors:** Emma L. Gunderson, Ian Vogel, Laura Chappell, Christina A. Bulman, K. C. Lim, Mona Luo, Jeffrey D. Whitman, Chris Franklin, Young-Jun Choi, Emilie Lefoulon, Travis Clark, Brenda Beerntsen, Barton Slatko, Makedonka Mitreva, William Sullivan, Judy A. Sakanari

**Affiliations:** 1 Dept. of Pharmaceutical Chemistry; University of California, San Francisco; San Francisco, California, United States of America; 2 Dept. of Molecular, Cell and Developmental Biology; University of California, Santa Cruz; Santa Cruz, California, United States of America; 3 Dept. of Laboratory Medicine; University of California, San Francisco; San Francisco, California, United States of America; 4 Division of Infectious Diseases; Washington University School of Medicine, St. Louis; St. Louis, Missouri, United States of America; 5 Molecular Parasitology Division; New England BioLabs; Ipswich, Massachusetts, United States of America; 6 Veterinary Pathobiology; University of Missouri-Columbia; Columbia, Missouri, United States of America; Pennsylvania State University, UNITED STATES

## Abstract

Antibiotic treatment has emerged as a promising strategy to sterilize and kill filarial nematodes due to their dependence on their endosymbiotic bacteria, *Wolbachia*. Several studies have shown that novel and FDA-approved antibiotics are efficacious at depleting the filarial nematodes of their endosymbiont, thus reducing female fecundity. However, it remains unclear if antibiotics can permanently deplete *Wolbachia* and cause sterility for the lifespan of the adult worms. Concerns about resistance arising from mass drug administration necessitate a careful exploration of potential *Wolbachia* recrudescence. In the present study, we investigated the long-term effects of the FDA-approved antibiotic, rifampicin, in the *Brugia pahangi* jird model of infection. Initially, rifampicin treatment depleted *Wolbachia* in adult worms and simultaneously impaired female worm fecundity. However, during an 8-month washout period, *Wolbachia* titers rebounded and embryogenesis returned to normal. Genome sequence analyses of *Wolbachia* revealed that despite the population bottleneck and recovery, no genetic changes occurred that could account for the rebound. Clusters of densely packed *Wolbachia* within the worm’s ovarian tissues were observed by confocal microscopy and remained in worms treated with rifampicin, suggesting that they may serve as privileged sites that allow *Wolbachia* to persist in worms while treated with antibiotic. To our knowledge, these clusters have not been previously described and may be the source of the *Wolbachia* rebound.

## Author summary

Onchocerciasis (river blindness) and lymphatic filariasis (elephantiasis) are two neglected tropical diseases caused by filarial nematodes, which harbor the endosymbiotic bacteria, *Wolbachia*. Major efforts to discover new drugs to treat these diseases have led to the discovery of novel compounds including those that target *Wolbachia*. We investigated the long-term effects of rifampicin on the filarial nematode, *Brugia pahangi*, and its endosymbiont, *Wolbachia*, in an *in vivo* rodent model of infection. Initially, *Wolbachia* titers were significantly reduced by 95% and female fecundity was impaired shortly after treatment. 8 months later however, *Wolbachia* rebounded and embryogenesis returned to normal. Sequence analysis of the *Wolbachia* genome revealed that despite the population bottleneck and recovery, no genetic changes occurred that could account for the rebound. Clusters of *Wolbachia* were observed within the ovaries of female worms throughout the entire 8-month study. These clusters may sequester *Wolbachia* and allow the bacteria to persist during antibiotic treatment, thereby enabling them to repopulate ovarian tissues and ensuring their vertical transmission to future generations of microfilariae.

## Introduction

Onchocerciasis, commonly known as river blindness, and lymphatic filariasis (LF), commonly known as elephantiasis, are neglected tropical diseases caused by filarial worms that together affect an estimated 86 million people worldwide [1]. Approximately 1.2 million people are visually impaired due to river blindness, while 12 million people with LF have complications due to elephantiasis [1]. River blindness is caused by the release of thousands of microfilariae (mf) from adult *Onchocerca volvulus* females residing in subcutaneous tissues. Mf migrate through the skin causing severe itching and skin depigmentation and also migrate to the ocular region where they induce an inflammatory response that can lead to blindness [2]. LF is caused by *Wuchereria bancrofti*, *Brugia malayi* and *B*. *timori* worms that reside in the lymphatic tissues where they cause tissue damage. While many infections are asymptomatic, individuals that develop the disfiguring disease often experience pain and severe lymphedema typically in the arms, legs, breasts and genitalia [2]. This can result in stigma associated with elephantiasis and extreme economic loss for individuals suffering from this disease [3]. The years lived with disability (YLDs) for LF and onchocerciasis are estimated to be 1.4 million and 1.3 million, respectively [1].

Current treatments primarily target the mf and not the adult worms, which are capable of surviving in their human host for 10–14 years for *O*. *volvulus* and 6–8 years for *Brugia* spp. [2,4–9]. For this reason, international control programs require annual or biannual mass drug administration of drugs in order to reduce transmission rates. However, given the longevity and high fecundity of these worms and the current lack of drugs that kill the adult worms, it is unlikely that the WHO goal of eliminating LF and onchocerciasis by 2030 will be met when microfilaricidal drugs are used alone [10–15]. The inability to reduce transmission rates with microfilaricides is compounded by the fact that ivermectin (IVM) cannot be distributed in areas co-endemic for another filarial nematode, *Loa loa*, due to the risk of severe adverse events, especially toxic encephalopathy when individuals are co-infected with high loads of *L*. *loa* mf [16,17].

*Onchocerca*, *Wuchereria* and *Brugia* spp., like many other species of filarial nematodes, harbor an intracellular endosymbiont, *Wolbachia*, which is important for female worm fecundity and survival [18–22]. *Loa loa*, however, lacks this bacterium, and efforts are underway to develop anti-*Wolbachia* drugs to eliminate this bacterium, thereby resulting in death of adult worms. In clinical trials conducted on patients with onchocerciasis and lymphatic filariasis, doxycycline was shown to deplete *Wolbachia* and eventually eliminate the adult worms after about 1–2 years [23,24]. Doxycycline however requires lengthy dosing regimens (100–200 mg daily for 4–6 weeks) and is therefore not practical for mass drug distribution. In addition, doxycycline is contraindicated in children 8 years and younger and because it is in pregnancy category D, should not be given to pregnant women [2].

Several studies have recently shown that short courses of 7- and 14-days of anti-*Wolbachia* compounds hold promise as excellent drugs to treat onchocerciasis and LF [25–29]. Studies by Hübner et al. however, showed that suboptimal treatment regimens of doxycycline in the *Litomosoides sigmodontis* infection model did not lead to a sustained reduction in *Wolbachia* loads in worms 14–18 weeks post-treatment and that longer term studies were needed to assess permanent sterilization of female adult worms [27,29]. Although West African cattle infected with *Onchocerca ochengi* are excellent hosts for long-term studies to evaluate the efficacy of antibiotics for the treatment of human onchocerciasis [30–36], the purpose of our study was to investigate the long-term effects of rifampicin in a rodent model of infection. In the present study, we investigated the use of jirds infected with *Brugia pahangi* to assess the effects of rifampicin on *Wolbachia* and worm survival in an 8-month time course study in which *Wolbachia* titers were determined using adult worms recovered from animals treated with a one-week dosing regimen of rifampicin.

## Results

### The endosymbiont *Wolbachia* rebound after 8 months following rifampicin treatment without genetic change

**Fig 1:**
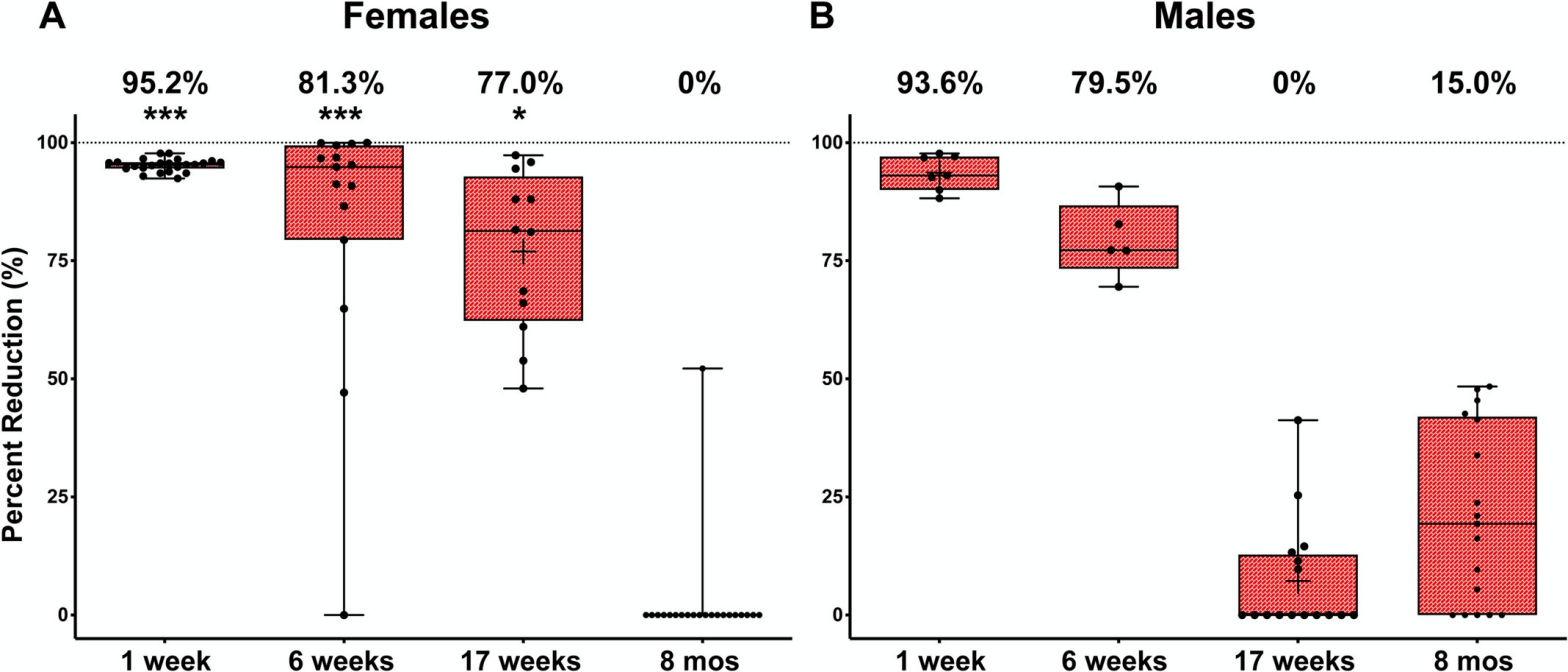
*Wolbachia* titers return to control levels 8 months after rifampicin treatment. Female and male *Brugia pahangi* from jirds treated with rifampicin were analyzed by qPCR to determine *Wolbachia* titers at each timepoint. Mean percent reductions of *Wolbachia wsp*/*gst* ratios from female (A) and male (B) adult worms are shown at 1-week, 6-weeks, 17-weeks and 8-months post-first dose. Data shown are medians and the boxes are the 25^th^ and 75^th^ percentiles with ***P<0.001 and *P<0.05. n = 2–9 jirds per treatment group per timepoint. Additional information is shown in S1 Table.

The research objective was to determine the long-term effects of antibiotic treatment on filarial worms and their endosymbiont, *Wolbachia*. We hypothesized that rifampicin would deplete worms of *Wolbachia* which would eventually lead to adult worm death in jirds infected with *Brugia pahangi*. *Wolbachia* titers in adult male and female worms were determined by qPCR at 1 week, 6 weeks, 17 weeks and 8 months post-first dose following the protocol by McGarry et al [37]. The relative abundance of single copy genes encoding the *Wolbachia* surface protein (*wsp*) were normalized to that of *Brugia* glutathione-S-transferase gene (*gst*) [28,29,38–40]. At the 1-week timepoint, *Wolbachia* titers were significantly reduced by 95.2% in female worms (Fig 1A). By 6 weeks however, the reduction in *Wolbachia* titers was reduced by 81.3% compared to those of control worms and by 17 weeks, titers were reduced by 77%. At 8 months, *Wolbachia* titers returned to levels similar to those of control worms, i.e. there was a 0% reduction in *Wolbachia* titers (Fig 1A). *Wolbachia* titers from male worms followed a similar trend of rebound (Fig 1B).

Although there was a significant effect of rifampicin on *Wolbachia* titers at early timepoints (S1 Table), rifampicin did not reduce the number of adult worms recovered at the time of necropsy at any of the timepoints, i.e. no macrofilaricidal effects were observed (S1 Fig).

To determine if the rebound in *Wolbachia* occurred as a result of genome changes (e.g. antibiotic resistance/tolerance), we sequenced *Wolbachia* genomes using hybridization probe-capture method from the treatment and vehicle groups at the 1-week and 8-month timepoints [41]. On average ~90,000 PacBio CCS reads were generated per sample, which amounts to ~150× coverage of the genome (S2 Table). A complete circularized reference genome (1,072,983 bp) was assembled using the 1-week vehicle group and sequence variants were identified in each group with respect to the reference. Variants occurring within the genomic regions likely representing nuclear *Wolbachia* transfers (nuwts [42]) were excluded from the analysis.

One single nucleotide variant (SNV) and five insertion/deletion variants (INDEL) were identified and their allele frequencies were estimated based on the number of reads that support each allele in each sample (S3 Table). The SNV (C-to-T substitution) occurred within the ORF of a short-chain dehydrogenase/reductase family (SDR) oxidoreductase and was predicted to be a synonymous variant and therefore a silent mutation. The allele frequency of this variant increased from 5.1% in the 1-week vehicle group to 35.5% in the 8-month rifampicin group (Fisher’s exact test P-value: 3.2×10^−5^), which was the only variant whose allele frequency displayed a statistically significant change. The INDEL variants invariably occurred within homopolymer regions (9–13 consecutive bases of A or T). Homopolymeric tracts are mutational hotspots because they are vulnerable to slippage errors during replication and transcription [43]. However, INDEL calling is error-prone around homopolymer runs (due to sequencing and PCR errors), and we cannot exclude the possibility that these INDELs are false-positive variants [44]. These data suggest that, despite the population bottleneck and recovery, the genetic change in *Wolbachia* after rifampicin treatment likely did not occur.

### Female worm fecundity is significantly reduced shortly after treatment but returns to control levels by 17 weeks

Commensurate with the depletion of *Wolbachia* 1 week after the first dose of rifampicin, we observed a significant impact on embryogenesis 6 weeks post-antibiotic treatment. This was followed by a gradual rebound in *Wolbachia* and return to normal embryogenesis by 17 weeks.

**Fig 2:**
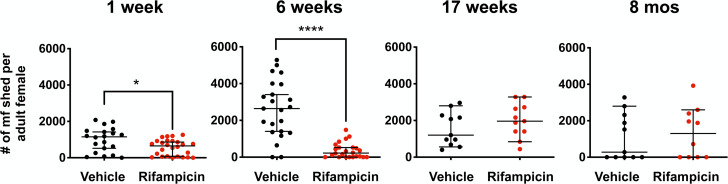
Rifampicin decreases mf shedding from female worms up to 6 weeks, followed by a return to normal by 17 weeks. The number of mf shed overnight by adult female worms that were recovered 1 week, 6 weeks, 17 weeks and 8-months post-first dose is shown for each timepoint. Mf shed overnight at the 1- and 6-week timepoint were significantly reduced (*P<0.05 and ****P<0.0001, respectively). Data are shown as median ± 95% CI. n = 10–26 female worms from n = 2–9 jirds per treatment group per timepoint.

The fecundity of female *B*. *pahangi* at each timepoint was assessed by counting the number of mf released after worms were removed from the animals and incubated *in vitro* for 18 hours. Worms from rifampicin treated jirds at the 1- and 6-week timepoint showed a significant reduction in the number of mf that were shed compared to worms from the vehicle group (43.4% reduction, P<0.05 and 86.3% reduction, P<0.0001, respectively) (Fig 2). Female fecundity returned to control levels after 17 weeks and remained at control levels for up to 8 months.

**Fig 3:**
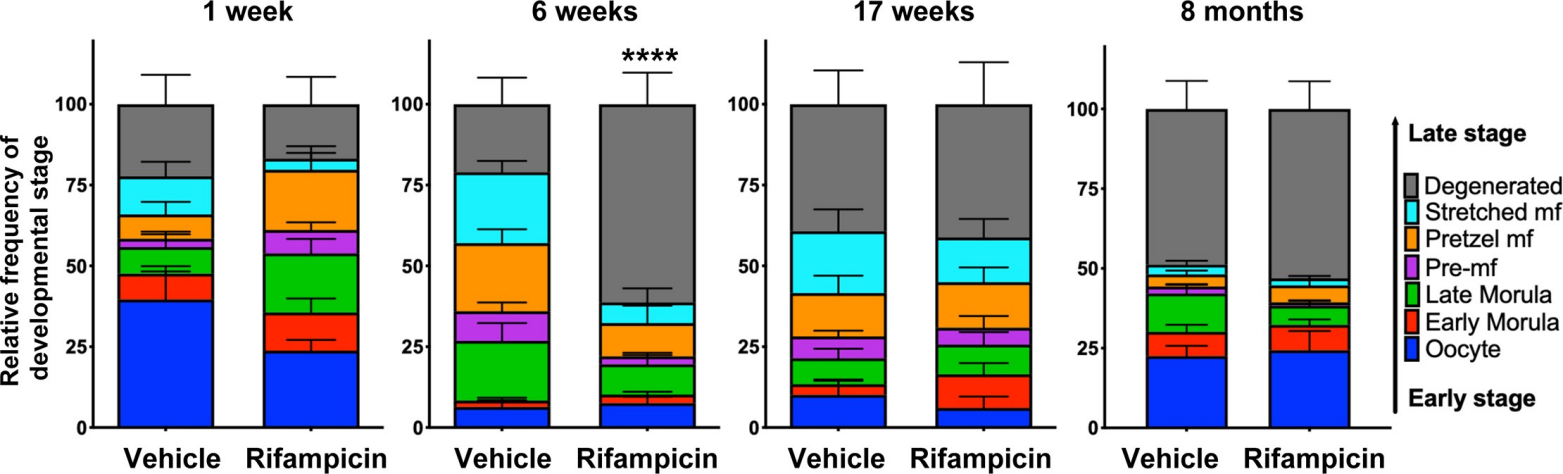
Rifampicin treatment leads to impaired embryogenesis by the 6-week timepoint but normal developmental stages return by 17 weeks. Embryonic stages found within the ovaries and uteri from female worms from the vehicle and treated groups were counted 1 week, 6 weeks, 17 weeks and 8 months post-first dose. There was a significant decrease in the frequency of healthy embryos across all developmental stages of embryogenesis at the 6-week timepoint (****P<0.0001). Percentages of degenerated embryos (gray) were also determined for each timepoint. Data are presented as mean ± SEM. n = 6–9 female worms from n = 2–9 jirds per treatment group per timepoint.

Embryograms of female worms were also analyzed to determine the effects of rifampicin on the developing stages of mf within the reproductive tract of female worms. Results showed that developmental stages from female worms recovered from rifampicin treated jirds at the 6-week timepoint exhibited a significant decrease in healthy embryos and an increase in degenerated embryos compared to those from both the control group at the 6-week timepoint, and the rifampicin group at the 1-week timepoint, where little disruption was observed (Fig 3 and S4 Table). The decrease in fecundity and disruption of embryogenesis however were not observed at later timepoints suggesting that embryonic development of mf returned to control levels following the rebound of *Wolbachia*.

### Cellular analysis reveals the rebound is derived from clusters of *Wolbachia*

As an independent method of analyzing *Wolbachia* titer, we performed fluorescence confocal analysis to image *Wolbachia* and host cell nuclei as previously described by Landmann et al., Serbus et al. and Foray et al. [45–49].

**Fig 4:**
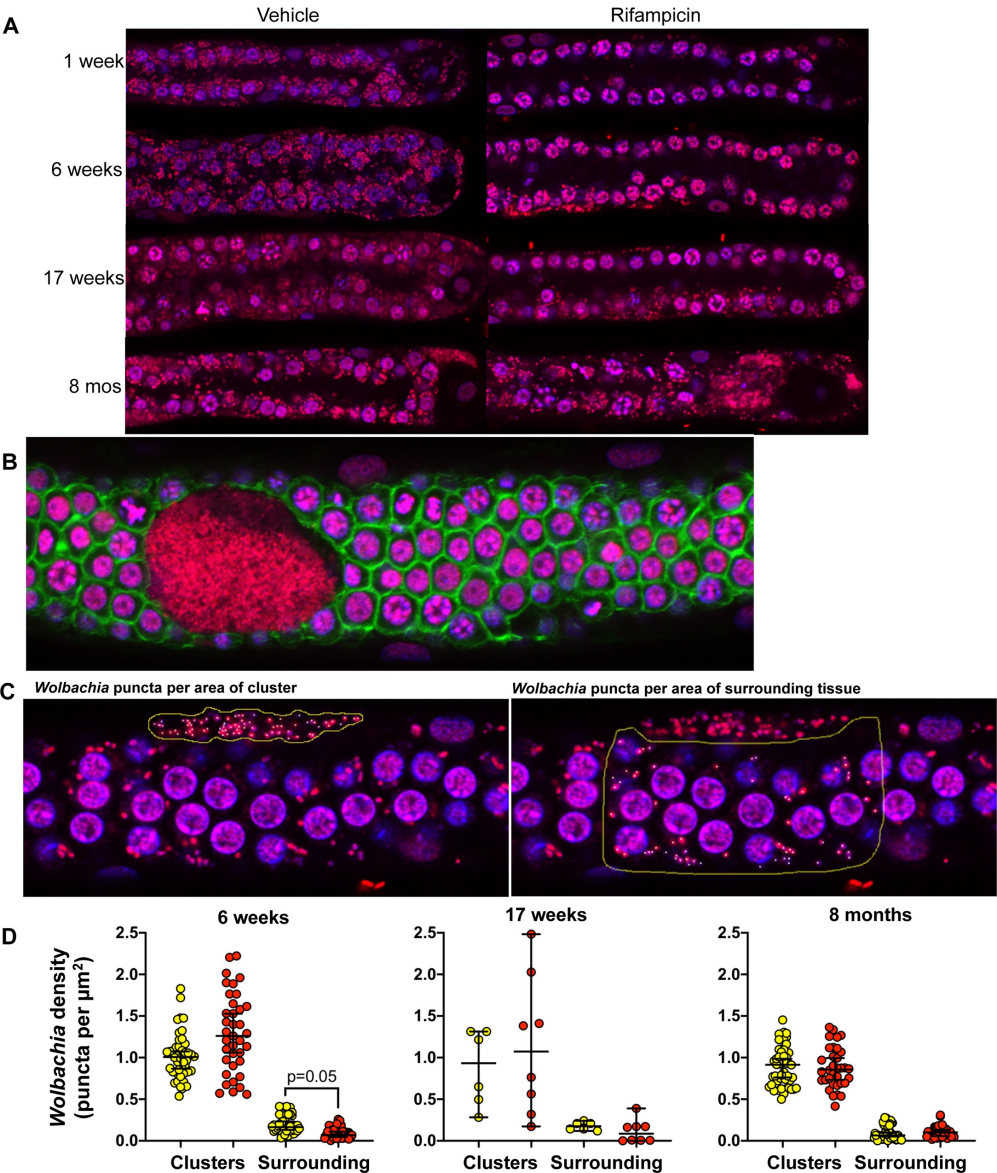
Rebound of *Wolbachia* at later timepoints may be driven by clusters. Ovaries were removed from individual female worms, fixed and stained for host nuclei (magenta) and *Wolbachia* (red). (A) *Wolbachia* are depleted at 1- and 6-weeks post-treatment but begin to rebound by 17 weeks. (B) Clusters of *Wolbachia* are seen in ovaries from vehicle and rifampicin worms; actin (green) stained with phalloidin; *Wolbachia* (red) stained with propidium iodide and host nuclei (magenta) stained with DAPI. (C) *Wolbachia* were quantified by counting number of puncta within the clusters (left) and in the periphery of the clusters (right). (D) Clusters were analyzed from worms collected at 6 weeks (n = 36–46), 17 weeks (n = 6–8) and 8 months (n = 34–52). Vehicle control worms in yellow and worms from rifampicin treated groups in red. Peripheral *Wolbachia* were reduced in females recovered from rifampicin treated jirds at the 6-week timepoint only (P = 0.05). Data are presented as median ± 95% CI.

Fluorescence imaging of the distal tip region of the ovaries revealed that *Wolbachia* were nearly depleted from germline tissues at the 1-week timepoint, but they began to increase in number at later timepoints (Fig 4A). Interestingly, large densely packed “clusters” of *Wolbachia* (Fig 4B) were observed in worms recovered from both the vehicle and treated groups at each timepoint. When *Wolbachia* were quantified in each of the clusters from ovaries (Fig 4C), results showed there were no significant differences between the treated and control groups with respect to the *Wolbachia* density (puncta per area of cluster, Fig 4D). However, *Wolbachia* in the peripheral areas around the clusters were significantly reduced (P = 0.05) in the worms from the rifampicin group at 6-weeks but not at the later timepoints (Fig 4D).

The presence of clusters with densely packed *Wolbachia* in ovaries from both rifampicin treated and control worms suggests that despite clearance of bacteria from the areas outside the clusters, the *Wolbachia* nevertheless persisted within the clusters and may be the source of the rebound. Analyses were not conducted on male worms as the focus of the work was on female fecundity and embryogenesis. We postulate that similar clusters will be associated with male worms, possibly in the hypodermal areas and, as with females, are likely responsible for the rebound.

## Discussion

Major efforts to identify new drugs to treat the adult stage (macrofilariae) of *Onchocerca volvulus* have led to the discovery of novel anti-*Wolbachia* compounds. These studies demonstrated that their respective anti-*Wolbachia* compounds significantly reduced *Wolbachia* titers in worms from animals treated with short courses of quinazolines and Tylosin analogs after 16–18 weeks post-first dose [26,27,29,40,50]. In addition, a one-week combination treatment of rifampicin and albendazole resulted in a greater than 99% reduction in *Wolbachia* levels in *B*. *malayi* infected SCID mice [28]. Clinical trials further revealed that albendazole either alone or in combination with antibiotics dramatically reduced *Wolbachia* levels [51,52]. With the recent discoveries of new anti-wolbachial drugs, we explored the *Wolbachia*/worm relationship *in vivo* to better understand the outcomes of antibiotic exposure. The aim of this study was to determine the longer-term effects of rifampicin on female worm survival and fecundity and *Wolbachia* titers using the *Brugia pahangi* infected jird model.

Although macrofilaricidal effects were not observed following treatment with rifampicin, *Wolbachia* titers and female fecundity were significantly affected 1 week post-first dose. *Wolbachia* titers were reduced by >95% in female worms from rifampicin treated animals at the 1-week timepoint compared to those of worms from vehicle animals. The extensive reduction of *Wolbachia* titers in adult worms has been previously described in other rodent studies with filarial worms, but the studies were terminated 16–18 weeks post-treatment [26,27,29,40,50], and the long-term effects of antibiotic treatment on filarial worms and their *Wolbachia* were not evaluated in these models.

Our present long-term study showed that even though *Wolbachia* were almost completely eliminated at early timepoints, *Wolbachia* rebounded to levels similar to those of control worms 8 months after rifampicin dosing. Female worms recovered from this timepoint were also fully reproductive, similar to female worms from the control group. Thus, despite qPCR data that showed a >95% reduction in *Wolbachia* titers, *Wolbachia* rebounded and returned to levels comparable to those of control worms, suggesting that this level of elimination is not sufficient to ensure permanent elimination and permanent female sterility. This finding parallels the rebound of *Wolbachia* titers in *O*. *ochengi* from cattle treated with a short, intensive regimen of oxytetracycline (10 mg/kg QD for 14 days) reported by Gilbert et al. [32], and suggests that this jird model is a suitable alternative for long-term studies (up to 8 months) in situations where cattle models cannot be conducted.

The dosage of rifampicin used in the present study was not likely suboptimal since previous studies with *B*. *malayi* infected SCID mice showed that 25 mg/kg rifampicin twice a day for 7 days resulted in reductions in *Wolbachia* comparable to the clinical dosage of doxycycline that caused >90% *Wolbachia* depletion [25]. While we saw the expected reduction in *Wolbachia* at early timepoints, the reduction was not sustained, and *Wolbachia* rebounded. Comparison of the genetic profiles of *Wolbachia* genomes from the early and late timepoints revealed that despite the population bottleneck and recovery, no genetic changes occurred in *Wolbachia* that could account for the rebound.

The significance of the rebound in this bacteria/worm symbiosis was evident when low titers of *Wolbachia*, which initially led to the reduction of late-stage embryos and microfilariae at 1- and 6- weeks, was later followed by the return of developing embryos and microfilariae released by female worms. This recovery back to control levels was commensurate with *Wolbachia* rebound, in line with evidence showing the dependence of female worms on *Wolbachia* to maintain their reproductive output [21,32,49,53–55].

Direct visualization of worm ovaries using fluorescence confocal microscopy revealed two distinct populations of *Wolbachia*: “clusters” of *Wolbachia* that were found within the female ovaries and “peripheral” *Wolbachia* that were found surrounding the clusters. For the *Wolbachia* within clusters, we found no significant differences in bacterial cluster amount, size, or density between rifampicin and control treatments, suggesting that these clusters do not respond to antibiotics, at least at the dosages used in this study. In contrast, we found that rifampicin significantly reduced the peripheral *Wolbachia* surrounding the clusters at 6 weeks. To our knowledge, these clusters have never before been identified in any *Wolbachia*-nematode system, and we believe that the clusters could serve as a reservoir of bacteria that can repopulate the germline tissue after antibiotic treatment.

Since genome sequencing of the rebounded *Wolbachia* showed there were no gene changes that could account for the persistence, we postulate that the clusters are privileged sites in which *Wolbachia* persist in a low or inactive metabolic state, similar to what occurs with intracellular *Toxoplasma gondii* bradyzoites [56,57] and pathogenic intracellular bacteria including *Mycobacterium tuberculosis*, *Treponema pallidum*, *Chlamydia* spp. and *Salmonella enterica* which can cause persistent and latent infections [58–66]. Interestingly, bacterial toxin-antitoxin (TA) genes of the *RelEB* family thought to cause persister cell formation in insect-associated *Wolbachia*, were absent in *Wolbachia* from filarial nematodes, suggesting that TAs may not be involved in persister formation of *Wolbachia* within the clusters and that other mechanisms are likely at play [67–70].

Although the molecular mechanisms of persister formation is not known in the *Wolbachia*-worm relationship, *Wolbachia* may respond in some manner to their low numbers and repopulate the ovarian tissues by moving from the clusters to the peripheral areas within the ovaries or by migrating into the worm’s pseudocoelom [46,71] and back into the ovaries, thereby ensuring their vertical transmission for future generations of microfilariae.

Further studies to define the nature of these clusters and wolbachial persistence will yield new information on the cell biology of this bacteria-worm symbiosis and may reveal similar strategies used by various pathogens that allow them to persist and remain latent within their hosts.

## Conclusion

We describe the effects of rifampicin treatment on the *Wolbachia*-*Brugia pahangi* relationship over an 8-month period in a rodent model. *Wolbachia* numbers were significantly reduced after initial treatment but subsequently rebounded along with a corresponding return of embryogenesis and fecundity in female worms. This *in vivo B*. *pahangi*/jird model serves as a useful tool to evaluate the long-term effects of antibiotics on *Wolbachia* depletion and female worm fecundity and provides information that may impact the clinical use of antibiotics to treat filarial diseases.

This study also provides insight into the *Wolbachia*-worm relationship with the discovery of two different populations of *Wolbachia* within the ovaries of these filarial worms: clusters of *Wolbachia* and peripheral *Wolbachia*, the former of which may account for the rebound of *Wolbachia* following antibiotic treatment. To our knowledge, clusters of *Wolbachia* have never before been identified in any *Wolbachia*-nematode system and may represent sequestered populations of this endosymbiont within *Brugia pahangi* ovaries.

## Methods

### Ethics statement

All animal studies were performed under the University of California, San Francisco Institutional Animal Care and Use Committee (IACUC) approvals AN109629-03 and AN173847-02 and adhered to the guidelines set forth in the NIH Guide for the Care and Use of Laboratory Animals and the USDA Animal Care Policies.

### Animal infections

For dosing studies on adult worms, male Mongolian jirds 50–60 grams, 5–7 weeks in age (*Meriones unguiculatus*, Charles River Laboratories International, Inc., Wilmington, MA) were injected intraperitoneally (IP) with third-larval stage *Brugia pahangi* (University of Missouri-Columbia) and treated 3 months later when larvae developed into adult worms.

### Drug dosages

Rifampicin (Research Products International Corp., Prospect, IL) was dissolved in 55% polyethylene glycol 400 (Sigma), 25% propylene glycol (Sigma), 20% water at a concentration of 5 mg/mL, and animals were given oral doses of 25 mg/kg twice a day for 7 days. We selected the dosage 25 mg/kg BID for 7 days based on findings from Aljayyoussi et al, 2017 which reported rifampicin dosages of 15 mg/kg QD for 7 days, 35 mg/kg QD for 7 days, or 25 mg/kg BID for 7 days resulted in reductions in *Wolbachia* of 97.7%, 98.2% and 99.5%, respectively [25]. They found 25 mg/kg BID x 7 days to be superior to doxycycline 25 mg/kg BID treatment for four weeks (P>0.0001) and not significantly inferior from doxycycline 25 mg/kg BID treatment for six weeks [25].

### PK analyses

To determine the level of exposure of rifampicin in treated animals, blood was collected from the saphenous vein in Am-heparinized tubes and centrifuged at 2,000 x g for 15 min at 4°C. Blood was collected 0.5 hour, 1 hour, 3 hours and 6 hours post-first dose. The second dose was given 8 hours post-first dose and blood was collected 24 hours post-first dose. Plasma was collected and stored at -80°C prior to shipment to Integrated Analytical Solutions (Berkeley, CA) for plasma analysis (S2 Fig). Calibration standards, QC samples and study samples were processed for LC/MS/MS analysis by precipitating 10 μL of each sample with 3 volumes of ice cold Internal Standard Solution (acetonitrile containing 50 ng/mL dextromethorphan). The precipitated samples were centrifuged at 6100g for 30 min and an aliquot of each supernatant was transferred to an auto sampler plate and diluted in water with 2 volumes of 0.2% formic acid.

### Necropsies

Vehicle and rifampicin treated jirds were necropsied 1 week, 6 weeks, 17 weeks and 8 months post-first dose. Animals were dissected and peritoneal cavities were washed with 100 mL of PBS to collect adult worms and mf that had been released from female worms. Adult worms were separated by sex, counted and processed for subsequent analyses. Mf from the peritoneal cavity were quantified by mixing peritoneal wash 9:1 (v/v) with 0.04% methylene blue:water and counted using an inverted microscope. Data from each timepoint are from three replicate studies, except for the 17-week timepoint, in which the data are from one set. All animals were euthanized at their intended timepoints and no adverse events were observed. However, one animal had ascites, but was otherwise healthy at the 8-month timepoint.

### qPCR analysis of *Wolbachia* in adult *Brugia pahangi*

Adult worms collected during necropsies were snap-frozen in a dry-ice and ethanol bath prior to storage at -80° C. gDNA was extracted from individual female worms or from 4–25 male worms using a DNEasy Blood & Tissue Kit (QIAGEN) according to the manufacturer’s instructions. Genomic DNA was quantified using a NanoDrop One^c^ (Thermo Fisher Scientific) and qPCR was performed using a Geneoecopia 2x All-in-One Master Mix (Cat #QP001-01) in a Bio-Rad CFX Connect RT-PCR thermocycler. The single copy gene, *Wolbachia* surface protein (*wsp*), was used to quantify *Wolbachia* titers and the single copy gene, glutathione-S-transferase (*gst*), was used to quantify *Brugia* titers following the protocol of McGarry et al. [37]. Primers used for qPCR were based on *wsp* forward: 5’-CCCTGCAAAGGCACAAGTTATTG-3’; *wsp* reverse: 5’-CGAGCTCCAGCAAAGAGTTTAATTT-3’; *gst* foward: 5’-GAGACACCTTGCTCGCAAAC-3’; *gst* reverse: 5’-ATCACGGACGCCTTCACAG-3’. For *gst* amplification the following cycles were used: 95°C for 15 minutes, followed by 36 cycles of denaturation at 94°C for 15 seconds, annealing at 55° C for 30 seconds and elongation at 72° C for 30 seconds. Melting curve analysis was conducted by heating to 95°C for 1 minute, annealing at 55°C for 30 seconds and heating to 97°C. For *wsp* amplification: samples were heated at 95° C for 15 minutes, followed by 40 cycles of denaturation at 94°C for 10 seconds, annealing at 55°C for 20 seconds and elongation at 72°C for 15 seconds. Melting curve analysis was conducted by heating to 95°C for 1 minute, annealing at 55°C for 30 seconds and heating to 97°C.

### *Wolbachia* genome sequencing

Total genomic DNA was extracted from *Brugia pahangi* worms recovered from 1-week and 8-month timepoints using DNeasy Blood & Tissue Kit (QIAGEN) following the manufacturer’s protocol and quantified using Qubit (Invitrogen). Genomic DNA was subjected to hybridization probe-capture (adaptation of the protocol of Geniez et al. and protocol of Lefoulon et al.) to enrich for *Wolbachia* DNA using biotinylated probe-baits and magnetic streptavidin beads [41,72]. Prior to capture, genomic DNA was sheared using NEBNext FSII for 30 min at 37° and ligated to NimbleGen SeqCap adapters (NEBNext Ultra II kit), followed by AMPure bead purification (0.9X), PCR amplification and purification through AMPure beads (0.9X). The barcoded samples were then pooled (~400 ng per sample) and hybridization of DNA with *Wolbachia* specific EZ library probes was performed according to SeqCap EZ HyperCap protocol v1.0 (NimbleGen). The captured DNA library was amplified by PCR, purified using AMPure bead (0.9X), and subjected to PacBio circular consensus sequencing (CCS). A reference *w*Bp genome was assembled with Canu v1.9 [73] from the control sample (1-week vehicle group) after the CCS reads were trimmed to remove residual adapter sequences using seqtk. To minimize assembly errors due to the presence of *B*. *pahangi* sequences derived from nuclear *Wolbachia* transfers (nuwts), a draft assembly was first generated using all available reads, and then reads (longer than 3 kb) that mapped to the assembly without clipping were collected using minimap v2.17 [74] and assembled to produce a second draft assembly, which was then circularized using Circlator [75]. Assembly errors were further corrected through manual curation and Pilon v1.23 [76]. Genome annotation was performed using PGAP [77] and DFAST v1.2.4 [78]. Genetic variants (SNPs and indels) were called across samples by DeepVariant [79] after non-clipped CCS reads were aligned to the *w*Bp reference genome using minimap2. The depth-of-coverage patterns of clipped reads (that span the junctions between nuwts and *B*. *pahangi* DNA) along the *Wolbachia* genome were exploited to infer the location of regions that show sequence similarity to nuwts using samtools [80] and sequana_coverage v0.7.1 [81]. Using VCFtools v0.1.17 [82], false-positive variants occurring within these putative nuwts regions were filtered and excluded from further analyses. Finally, SnpEff v4.3 [83] was used to annotate and predict the effects of the remaining variants. The statistical significance of allele frequency differences was determined with Fisher's exact test.

### Embryograms and microfilariae overnight shed

Individual adult female worms (n = 12–24 females per treatment group) from vehicle and rifampicin treated jirds were maintained overnight in 24-well plates with 500 μL of RPMI-1640, 25mM HEPES, 5% heat inactivated-FBS, and 1x Antibiotic/Antimycotic. Mf that were released from individual females after 18 hours were removed from the wells and counted. For the embryogram analyses, individual adult female worms previously frozen in 0.5mL of 0.1% PBS-Triton X-100 (Sigma) were homogenized using a glass pestle to disrupt the cuticle and expose the reproductive structures. Developing stages from the ovaries and uteri (oocyte, early morula, late morula, pre-mf, pretzel mf and stretched mf, degenerated embryos) were assessed in a blinded fashion for their developmental stages using an inverted microscope and hemocytometer. A minimum of 100 developmental stages were counted from each female and relative proportions were used to determine the means and standard deviations. n = 6–9 females per treatment group per timepoint [45].

### Fluorescence imaging of female worm ovaries

Female *B*. *pahangi* worms removed from jirds from each group were frozen and shipped to UC Santa Cruz for fluorescence analysis. Frozen worms were thawed at room temperature, immediately fixed in 3.2% paraformaldehyde for 25 minutes and rinsed twice in PBST (PBS plus 0.1% Triton-X100). Individual female uteri/ovaries were dissected from fixed tissue and incubated overnight in RNAse A (10mg/mL) in PBST following similar protocols described by Landmann et al., Serbus et al. and Foray et al. [45–49]. Tissues were then stained with propidium iodide (PI) (1mg/mL diluted 100X in PBST) for 30 seconds, rinsed twice in PBST and then mounted in DAPI Vectashield mounting medium (Vector Labs). The distal tips of the ovarian tissue were imaged on a Leica SP5 confocal microscope and single images were taken at the mid-plane of the ovarian tissue for each distal tip. To measure the average *Wolbachia* titer of each distal tip, *Wolbachia* puncta were counted by hand using the Cell Counter tool in FIJI; the number of puncta was divided by the area of the tissue in each image (puncta/μm^2^).

### Statistical analyses

Data were first tested for normality using the Shapiro-Wilk test of normality. When data did not pass the normality test, a Mann-Whitney U test was conducted and when data did pass the normality test, a Student’s t-test was used. All significance levels were determined as compared to the vehicle worms at the same timepoint. Individual percent reductions of *Wolbachia* were calculated for each worm using the *wsp* copy number normalized to the *Brugia gst* copy number [28,29,38–40]. *Wsp/gst* ratios were calculated for each timepoint by subtracting the *wsp/gst* ratios of treated worms from the mean *wsp/gst* ratio of vehicle worms and then dividing by the mean *wsp/gst* ratio of vehicle worms for each respective timepoint. The means were then calculated to determine the average percent reduction. All statistical analyses were determined using Prism 8 version 8.2.0 (272).

## Supporting information

S1 Fig Rifampicin treatment of *Brugia pahangi*-infected jirds does not reduce worm burden significantly at any timepoint. (A) Number of total worms recovered from the peritoneal cavity of *B*. *pahangi*-infected jirds 1-week, 6-weeks, 17-weeks and 8-months post-first dose. (B) Number of female worms recovered from the peritoneal cavity of *B*. *pahangi*-infected jirds 1-week, 6-weeks, 17-weeks and 8-months post-first dose. (C) Number of male worms recovered from the peritoneal cavity of *B*. *pahangi*-infected jirds 1-week, 6-weeks, 17-weeks and 8-months post-first dose. Data is presented as median ± 95% CI. n = 2–9 jirds per treatment group per timepoint.
(PDF)

S2 Fig Plasma concentrations peak 3 hours post-rifampicin treatment of *Brugia pahangi*-infected jirds. Plasma samples were collected at 0.5, 1, 3, 6 and 24 hours post-first dose from *B*. *pahangi* infected jirds treated with oral doses of rifampicin 25 mg/kg twice a day for 7 days. Analyses showed that plasma concentrations peaked at 3 hours post-first dose with a C_max_ of 5.17x10^3^ ng/mL. n = 3 jirds for all timepoints except n = 2 at 6 hours and n = 4 at 24 hours.
(PDF)

S1 Table Results of *Wolbachia* titers from 3 experimental replicates after rifampicin treatment. qPCR analyses used worms from 3 separate cohorts of animals with data from two replicate experiments for each timepoint, except for the 17-week timepoint. Data are presented as mean percent reductions based on control worms.
(PDF)

S2 Table PacBio sequencing of *Wolbachia* using hybridization probe-capture. (PDF)

S3 Table Genetic variants identified in *Wolbachia* after rifampicin treatment. (PDF)

S4 Table Female worms recovered from jirds treated 6 weeks post-first dose had significantly higher numbers of degenerated embryos compared to those from the vehicle group. The number in each cell is the mean of each embryonic stage ± the standard deviation.
(PDF)
