# Supplementary figures and images for: The endosymbiont Wolbachia rebounds following antibiotic treatment
Source: PLoS Pathog. 2020 Jul 8;16(7):e1008623. doi: 10.1371/journal.ppat.1008623 (PMC7371230; doi:10.1371/journal.ppat.1008623)

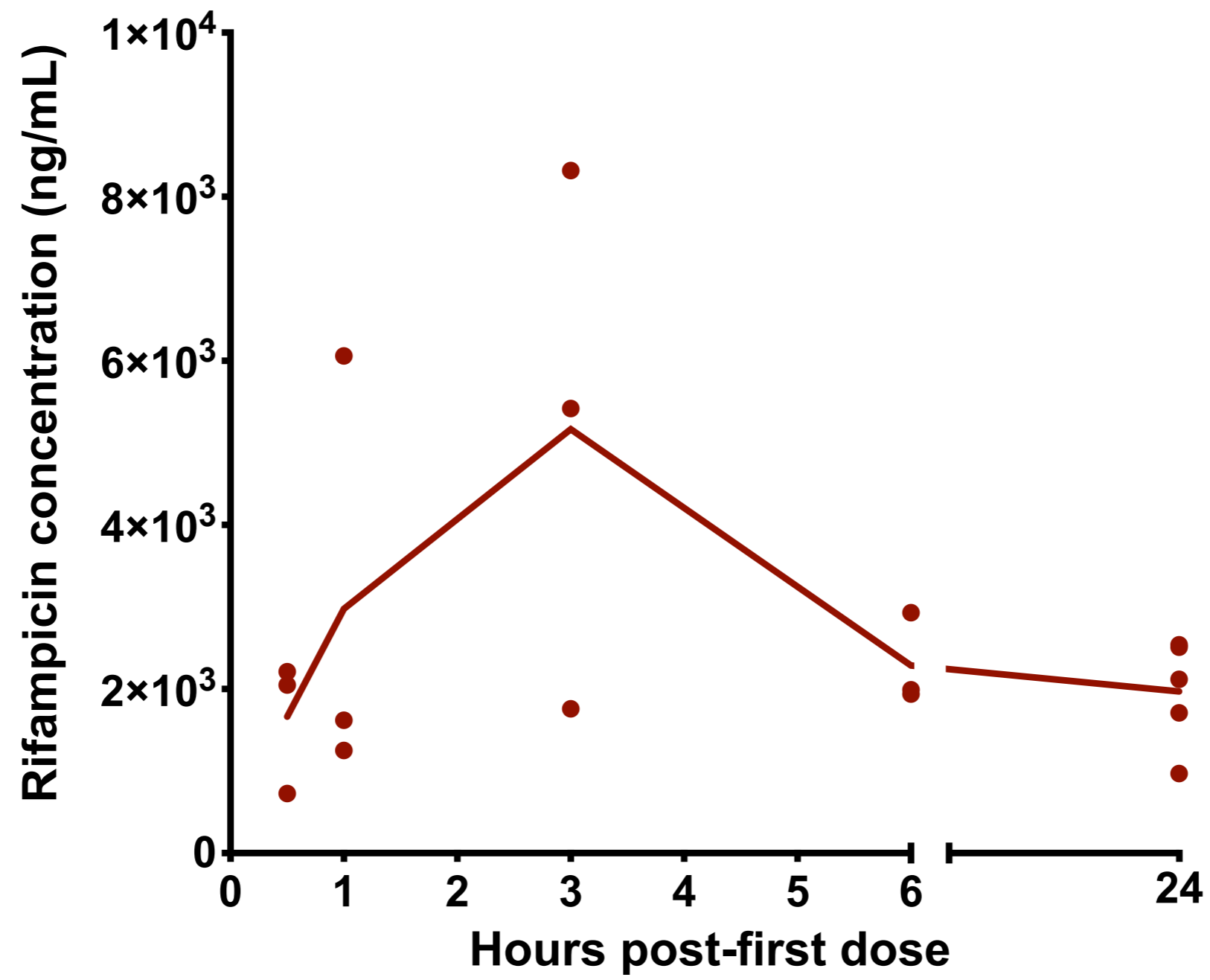

Supplement: S1 Fig — (A) Number of total worms recovered from the peritoneal cavity of B. pahangi-infected jirds 1-week, 6-weeks, 17-weeks and 8-months post-first dose. (B) Number of female worms recovered from the peritoneal cavity of B. pahangi-infected jirds 1-week, 6-weeks, 17-weeks and 8-months post-first dose. (C) Number of male worms recovered from the peritoneal cavity of B. pahangi-infected jirds 1-week, 6-weeks, 17-weeks and 8-months post-first dose. Data is presented as median ± 95% CI. n = 2–9 jirds per treatment group per timepoint. (PDF) [file ppat.1008623.s001.pdf]

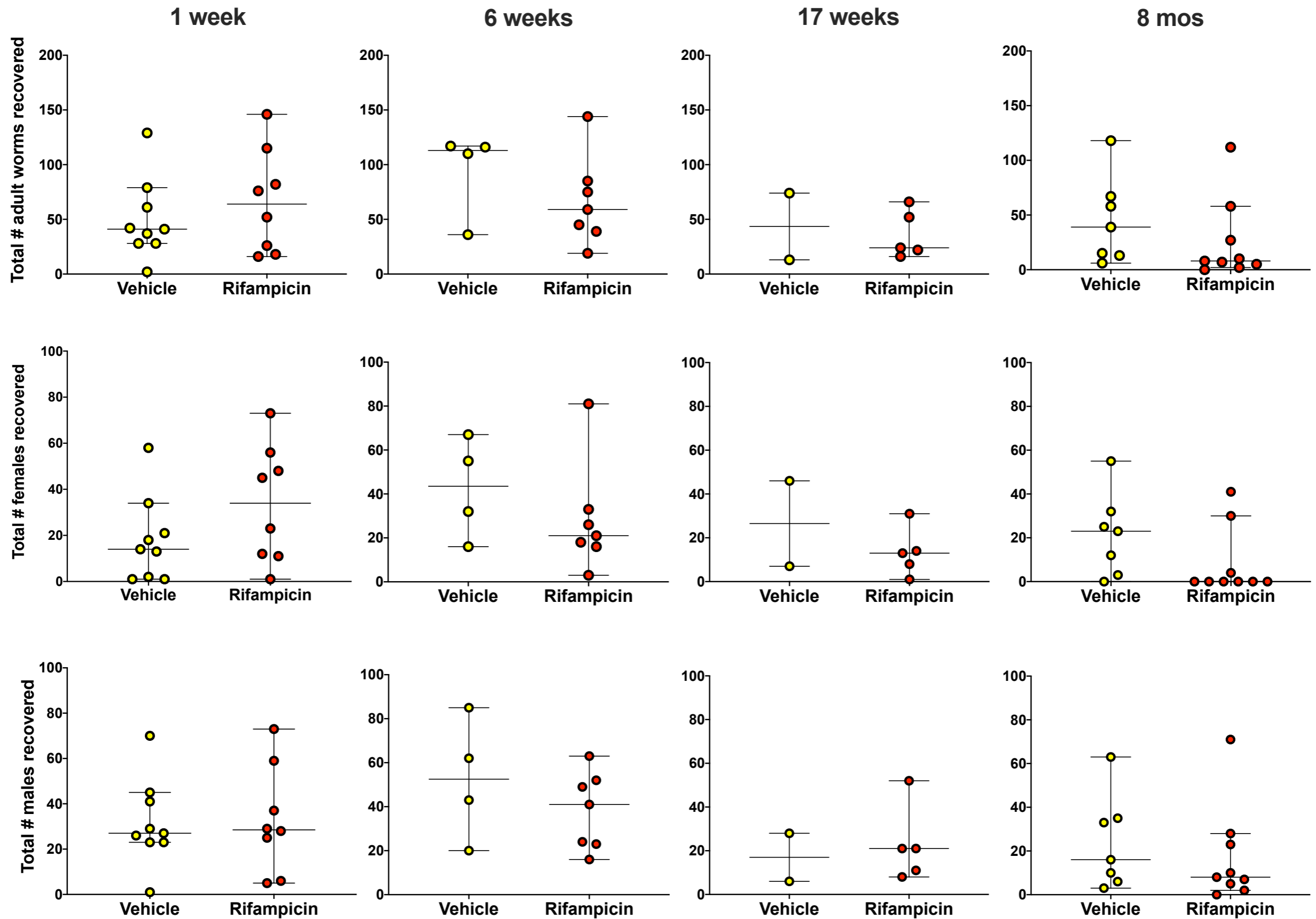

Supplement: S2 Fig — Plasma samples were collected at 0.5, 1, 3, 6 and 24 hours post-first dose from B. pahangi infected jirds treated with oral doses of rifampicin 25 mg/kg twice a day for 7 days. Analyses showed that plasma concentrations peaked at 3 hours post-first dose with a Cmax of 5.17x10^3^ ng/mL. n = 3 jirds for all timepoints except n = 2 at 6 hours and n = 4 at 24 hours. (PDF) [file ppat.1008623.s002.pdf]
