# Supplementary material for: The endosymbiont Wolbachia rebounds following antibiotic treatment
Source: PLoS Pathog. 2020 Jul 8;16(7):e1008623. doi: 10.1371/journal.ppat.1008623 (PMC7371230; doi:10.1371/journal.ppat.1008623)
Supplement: S1 Table — qPCR analyses used worms from 3 separate cohorts of animals with data from two replicate experiments for each timepoint, except for the 17-week timepoint. Data are presented as mean percent reductions based on control worms. (PDF) [file ppat.1008623.s003.pdf]

Timepoint and Replicates	# of jirds	*Wolbachia* percent reduction in female worms	# female worms	*wsp/gst* ratio female P value	*Wolbachia* percent reduction in male worms	# of pooled samples (total # male worms)	*wsp/gst* ratio male P value
**1 week**							
Replicate 1	3	95.70%	10	P<0.0001 (****)	96.90%	2 (60)	P=0.3333 (ns)
Replicate 2	5	94.80%	13	P<0.0001 (****)	92.30%	5 (20)	P=0.0079 (**)
**6 weeks**							
Replicate 1	3	86.90%	10	P<0.0001 (****)	83.90%	2 (55)	P=0.3333 (ns)
Replicate 2	4	70.10%	5	P=0.732 (ns)	76.50%	3 (12)	P=0.2000 (ns)
**17 weeks**							
Date set 1	5	77.00%	12	P=0.284 (*)	0%	16 (64)	P=0.4376 (ns)
**8 months**							
Replicate 1	1	0%	4	P=0.1419 (ns)	0%	3 (12)	P=0.2619 (ns)
Replicate 2	8	0%	17	P=0.0244 (*)	24.80%	14 (56)	P=0.0225 (*)

S1 Table. Results of *Wolbachia* titers from 3 experimental replicates after rifampicin treatment. qPCR analyses used worms from 3 separate cohorts of animals with data from two replicate experiments for each timepoint, except for the 17-week timepoint. Data are presented as mean percent reductions based on control worms.
