# Supplementary material for: The endosymbiont Wolbachia rebounds following antibiotic treatment
Source: PLoS Pathog. 2020 Jul 8;16(7):e1008623. doi: 10.1371/journal.ppat.1008623 (PMC7371230; doi:10.1371/journal.ppat.1008623)
Supplement: S2 Table — (PDF) [file ppat.1008623.s004.pdf]

Treatment	PacBio CCS reads			Reads mapped to wBp reference	
	Total read counts	Avg length (bp)	Total bases	Total bases	Mapping rate
Vehicle, 1 week	115,719	1,720	199,050,566	180,995,803	90.9%
Rifampicin, 1 week	98,687	1,728	170,506,464	146,231,064	85.8%
Vehicle, 8 months	95,501	1,779	169,927,794	154,341,209	90.8%
Rifampicin, 8 months	53,649	1,501	80,548,072	57,570,343	71.5%

S2 Table. PacBio sequencing of *Wolbachia* using hybridization probe-capture.
