# Supplementary material for: The endosymbiont Wolbachia rebounds following antibiotic treatment
Source: PLoS Pathog. 2020 Jul 8;16(7):e1008623. doi: 10.1371/journal.ppat.1008623 (PMC7371230; doi:10.1371/journal.ppat.1008623)
Supplement: S3 Table — (PDF) [file ppat.1008623.s005.pdf]

Position	Variant type	Reference allele	Alternate allele	Predicted effect	Affected gene	Homopolymer INDEL	Vehicle, 1 wk	Rifampicin, 1 wk		Vehicle, 8 mos		Rifampicin, 8 mos	
							Alternate allele frequency	Alternate allele frequency	P-value	Alternate allele frequency	P-value	Alternate allele frequency	P-value
218,175	INDEL	AT	A	Frameshift variant	50S ribosomal protein L33	Poly-T 9-mer	15.5%	13.4%	7.3E-01	12.1%	4.9E-01	18.6%	6.4E-01
252,600	SNV	C	T	Synonymous variant	SDR family oxidoreductase		5.1%	8.2%	4.0E-01	5.8%	1.0E+00	35.5%	3.2E-05
300,458	INDEL	TA	T	Intergenic region		Poly-A 10-mer	23.7%	24.6%	8.9E-01	23.4%	1.0E+00	10.9%	5.1E-02
433,559	INDEL	GA	G	Frameshift variant	50S ribosomal protein L23	Poly-A 13-mer	44.2%	41.4%	6.1E-01	47.5%	5.5E-01	30.8%	6.2E-02
770,141	INDEL	CA	C	Intergenic region		Poly-A 11-mer	35.3%	33.3%	7.9E-01	30.9%	5.0E-01	35.5%	1.0E+00
854,094	INDEL	TA	T	Frameshift variant	zinc ABC transporter solute-binding protein	Poly-A 10-mer	18.7%	27.2%	2.1E-01	12.8%	3.1E-01	26.3%	5.3E-01

S3 Table. Genetic variants identified in *Wolbachia* after rifampicin treatment.
