# Supplementary material for: The endosymbiont Wolbachia rebounds following antibiotic treatment
Source: PLoS Pathog. 2020 Jul 8;16(7):e1008623. doi: 10.1371/journal.ppat.1008623 (PMC7371230; doi:10.1371/journal.ppat.1008623)
Supplement: S4 Table — The number in each cell is the mean of each embryonic stage ± the standard deviation. (PDF) [file ppat.1008623.s006.pdf]

	1 week		6 weeks		17 weeks		8 mos	
	Vehicle	Rifampicin	Vehicle	Rifampicin	Vehicle	Rifampicin	Vehicle	Rifampicin
Oocyte	39.63 ± 21.14	23.79 ± 8.11	6.39 ± 5.04	7.57 ± 4.81	10.11 ± 10.63	6.04 ± 8.68	22.53 ± 9.62	24.33 ± 18.15
Early Morula	7.97 ± 5.60	11.82 ± 10.56	1.97 ± 2.22	2.65 ± 2.11	3.29 ± 3.58	10.44 ± 8.45	7.60 ± 6.55	7.95 ± 5.20
Late Morula	8.23 ± 11.69	18.27 ± 10.90	18.47 ± 13.46	9.36 ± 6.97	8.04 ± 7.23	9.20 ± 9.42	12.01 ± 8.82	6.03 ± 5.11
Pre-Mf	2.58 ± 3.36	7.23 ± 5.78	9.14 ± 6.62	2.48 ± 2.61	6.75 ± 4.35	5.18 ± 8.88	2.16 ± 1.66	1.04 ± 1.27
Pretzel Mf	7.51 ± 9.53	18.6 ± 17.92	21.08 ± 10.47	10.28 ± 13.24	13.42 ± 13.13	14.08 ± 11.27	3.83 ± 3.55	5.32 ± 5.96
Stretched Mf	11.79 ± 11.00	3.44 ± 4.38	21.91 ± 8.60	6.38 ± 10.67	19.13 ± 16.51	13.87 ± 14.02	3.05 ± 3.67	2.22 ± 2.27
Degenerated	22.29 ± 22.32	16.84 ± 20.71	21.05 ± 20.13	**61.3 ± 23.89 P<0.0001**	39.27 ± 25.64	41.2 ± 31.8	48.83 ± 26.37	53.11 ± 26.04

S4 Table. Female worms recovered from jirds treated 6 weeks post-first dose had significantly higher numbers of degenerated embryos compared to those from the vehicle group. The number in each cell is the mean of each embryonic stage ± the standard deviation.
